# KDM4B promotes acute myeloid leukemia associated with AML1‐ETO by regulating chromatin accessibility

**DOI:** 10.1096/fba.2021-00030

**Published:** 2021-09-12

**Authors:** Takeshi Ueda, Akinori Kanai, Akiyoshi Komuro, Hisayuki Amano, Kazushige Ota, Masahiko Honda, Masahito Kawazu, Hitoshi Okada

**Affiliations:** ^1^ Department of Biochemistry Kindai University Faculty of Medicine Osakasayama Japan; ^2^ Graduate School of Medical Sciences Kindai University Faculty of Medicine Osakasayama Japan; ^3^ Department of Molecular Oncology Research Institute for Radiation Biology and Medicine Hiroshima University Hiroshima Japan; ^4^ Division of Cellular Signaling National Cancer Center Research Institute Tokyo Japan; ^5^ Anti‐Aging Center Kindai University Higashi‐Osaka Japan

**Keywords:** acute myeloid leukemia, chromatin accessibility, gene expression analysis, gene targeting

## Abstract

Epigenetic alterations of chromatin structure affect chromatin accessibility and collaborate with genetic alterations in the development of cancer. Lysine demethylase 4B (KDM4B) has been identified as a JmjC domain‐containing epigenetic modifier that possesses histone demethylase activity. Although recent studies have demonstrated that KDM4B positively regulates the pathogenesis of multiple types of solid tumors, the tissue specificity and context dependency have not been fully elucidated. In this study, we investigated gene expression profiles established from clinical samples and found that *KDM4B* is elevated specifically in acute myeloid leukemia (AML) associated with chromosomal translocation 8;21 [t(8;21)], which results in a fusion of the *AML1* and the eight‐twenty‐one (*ETO*) genes to generate a leukemia oncogene, AML1‐ETO fusion transcription factor. Short hairpin RNA‐mediated KDM4B silencing significantly reduced cell proliferation in t(8;21)‐positive AML cell lines. Meanwhile, KDM4B silencing suppressed the expression of AML1‐ETO‐inducible genes, and consistently perturbed chromatin accessibility of AML1‐ETO‐binding sites involving altered active enhancer marks and functional cis‐regulatory elements. Notably, transduction of murine KDM4B orthologue mutants followed by KDM4B silencing demonstrated a requirement of methylated‐histone binding modules for a proliferative surge. To address the role of KDM4B in leukemia development, we further generated and analyzed *Kdm4b* conditional knockout mice. As a result, *Kdm4b* deficiency attenuated clonogenic potential mediated by AML1‐ETO and delayed leukemia progression *in vivo*. Thus, our results highlight a tumor‐promoting role of KDM4B in AML associated with t(8;21).

## 1 INTRODUCTION

Epigenetic patterns are required in maintaining cell identity, and their deregulation is associated with a variety of human diseases including cancer.^1^ The post‐translational modification of histones is an important epigenetic mechanism for modulating nucleosome dynamics to alter chromatin structure and for regulating gene expression.^2^ The lysine demethylase 4 (KDM4) family proteins, which consist of KDM4A−KDM4D, are JmjC domain‐containing epigenetic regulators that possess demethylase activity for di‐ and tri‐methylated histone H3 Lys9 (H3K9), as well as di‐ and tri‐methylated H3K36 and H1.4K26.^3^ In addition to the JmjC histone demethylase domain, KDM4A, KDM4B, and KDM4C contain double plant homeodomain (PHD), and Tudor domains as methylated‐histone binding modules.^3^ In contrast, KDM4D has been found to be a shorter protein that does not contain any of the PHD and Tudor domains.^3^ These KDM4A−KDM4D proteins were reported to be involved in the pathogenesis of solid tumors, and we previously identified a tumor‐promoting role of KDM4B in estrogen receptor‐positive breast cancer.^3^, ^4^

In addition to solid tumors, recent studies have indicated that epigenetic regulation significantly contributes to the pathogenesis of hematologic malignancies, including myeloid diseases.^5^, ^6^ The chromosomal translocation 8;21 [t(8;21)] is the most common karyotypic abnormality in acute myeloid leukemia (AML). The t(8;21) results in a fusion of the acute myeloid leukemia 1 (*AML1*)/Runt‐related transcription factor 1 (*RUNX1*) and the eight‐twenty‐one (*ETO*)/RUNX1 translocation partner 1 (*RUNX1T1*) genes to generate the *AML1*‐*ETO* (*AE*) fusion gene.^7^ The wild‐type (WT) AML1 protein has been determined as a key transcription factor for hematopoiesis,^8^ whereas the resulting gene fusion product (AE) functions as an oncoprotein. It contains the N‐terminal region of AML1, including the Runt DNA‐binding domain, but not the transactivation region, and almost the entire region of ETO, which was initially reported to function as a nuclear protein that interacts with transcriptionally repressive complexes.^7^ Thus, the chimeric AE protein has been reported to primarily function as a dominant negative form of AML1, but it can also induce gene expression via recruiting nuclear co‐activators, according to later studies.^7^ Consequently, the AE protein is able to enhance self‐renewal and retain undifferentiated properties in hematopoietic cells by altering the expression of multiple AML1 target genes, and contributes as a driver hit in promoting AML development.

While the post‐translational modifications of AE and/or its interaction with cofactors regulate the transcriptional activity, multiple epigenetic mechanisms including histone modifications and chromatin remodeling also participate in AE‐targeted gene expression.^7^ In addition, although the AE protein has the intact Runt DNA‐binding domain, its DNA‐binding properties are slightly different from those of native AML1, due to the altered DNA‐binding efficiency and cofactor dependency.^9^, ^10^ Accordingly, the genome‐wide distributions of AE and native AML1 are not identical, which leads to a selective preference of their DNA target sites.^7^, ^9^, ^10^ Also in turn, transcription factors themselves including AE could induce chromatin modifications.^11^ Clinically, this cytogenetic subset of AML is considered to have a relatively good prognosis; however, relapse occurs in up to 40% of patients even following an intensive chemotherapy.^12^, ^13^ In this study, we report that KDM4B promotes the pathogenesis of AE‐induced leukemia in human cell lines and a mouse model, and highlight the leukemia‐promoting role of KDM4B.

## 2 MATERIALS AND METHODS

### 2.1 Cell lines

The SKNO‐1, Kasumi‐1, and U937 cell lines were obtained from the Japanese Collection of Research Bioresources (JCRB) Cell Bank. The KG‐1a cell line was sourced from the RIKEN cell bank. SKNO‐1 was maintained in RPMI 1640 media containing 10% serum and 10 ng/ml human granulocyte‐macrophage colony‐stimulating factor (GM‐CSF; Peprotech). Kasumi‐1, U937, and KG‐1a were cultured in RPMI 1640 containing 10% serum.

### 2.2 RNA interference

For KDM4B knockdown, the pLKO.1 puro (Addgene plasmid #8453)‐based lentiviral shRNA vector was used. The target sequences of KDM4B sh#1 and sh#2 were shown previously.^4^ Scrambled shRNA (Addgene plasmid #1864) was used as a control. pLKO.1 puro‐BRG1 sh#1 and sh#2 (TRCN0000015549 and TRCN0000015552) were obtained from Horizon Discovery. Lentivirus was prepared as previously described,^4^ and then the lentivirus particles were concentrated using Lenti‐X Concentrator (Clontech).

### 2.3 Cell proliferation and viability assays

For knockdown experiments, lentivirus‐transduced cells were grown in 1.5 µg/ml puromycin containing media for 2 days. Subsequently, Kasumi‐1 and SKNO‐1 cells were seeded at 5 × 10^4^ or 1 × 10^5^ cells (typically 2 ml) per well in a 12‐well plate. The KG‐1a and U937 cells were plated at 1 × 10^4^ and 2 × 10^3^ cells, respectively, per well in a 12‐well plate. For add‐back experiments, the KDM4B‐silenced SKNO‐1 overexpressing murine WT or mutated KDM4B protein series were seeded at 1 × 10^5^ cells in a 12‐well plate. GM‐CSF was added at 10 ng/ml with 0.15 ml media for SKNO‐1 and cells were dispersed by pipetting in culture to avoid cell aggregation every 7 days post‐seeding. All experiments were performed in triplicate. Cell number and viability were determined by dye exclusion trypan blue assay using Countess (Life Technologies). Annexin V/propidium iodide staining was performed according to the standard method.

### 2.4 Plasmid construction and Establishment of Kdm4b mutant‐expressing cells

The mutated murine *Kdm4b* cDNAs used in this study were generated using polymerase chain reaction (PCR)‐based site‐directed mutagenesis. A triple FLAG tag sequence was introduced after the ATG starting codon of the WT *Kdm4b* and mutant series. The cDNAs were then cloned into CSII‐CMV‐MCS‐IRES2‐Bsd lentiviral vector (kindly provided by Dr. Miyoshi, RIKEN BRC). Lentivirus‐transduced SKNO‐1 cells were grown in 10 µg/ml blasticidin S containing media for 4 days and then used for further experiments.

### 2.5 RNA‐sequencing

Total RNAs were extracted using RNeasy Kit (Qiagen) and then converted into libraries using the Illumina TruSeq RNA sample preparation kit or Agilent SureSelect strand‐specific RNA library prep kit. Transcriptome analysis was performed using a next‐generation sequencer (HiSeq 2500; Illumina), according to the manufacturer’s instructions, and then the generated sequence tags were mapped onto the human genomic sequence (hg38) or mouse genomic sequence (mm10) using the Tophat/Cufflinks.

### 2.6 Gene expression data

We compared the gene expression levels from two different genotypes (KDM4B sh#1 and sh#2 vs. SCR, Kdm4b^Δ/Δ^ vs. Ctrl) for gene set enrichment analysis (GSEA).^14^ GSEA was performed by computing overlaps with previously published gene expression data^15^ ^–^ ^17^ and MYC1_Q2 from MSigDB. The false discovery rate (FDR) *q*‐value < 0.25 was considered significant.

### 2.7 Assay for transposase‐accessible chromatin sequencing (ATAC‐seq)

ATAC‐seq was performed as described previously.^18^ Nuclear extracts were prepared from 50,000 cells and then incubated with 2.5 µl of transposase (Nextera Tn5 Transposase; Illumina, Cat #FC‐121–1030) in a 50 µl reaction for 30 min at 37℃. After purification of transposase‐fragmented DNA, the library was amplified by PCR and was further subjected to high‐throughput sequencing using the HiSeq 2500 platform (Illumina). Accessible chromatin sites were identified as peaks using MACS2.^19^ The alignment of published ChIP‐seq signals for SKNO‐1 cell line^20^, ^21^ on accessible chromatin regions was done using the ngs.plot tool.^22^ The enrichment analysis of cis‐regulatory regions was done using the Genomic Regions Enrichment of Annotations Tool (GREAT).^23^

### 2.8 Mice

*Kdm4b^flox^* ^/^ *^flox^* and *Mx1*‐*Cre* mice [B6.Cg‐Tg(Mx1‐cre)1Cgn/J] have been described previously.^4^, ^24^ Cre activation was achieved by intraperitoneal administration of 500 µg of polyinosinic‐polycytidylic acid (pIpC; Sigma‐Aldrich) thrice at 2‐day intervals. Age‐ and gender‐matched littermates were used for all experiments 3 weeks after the final pIpC injection unless otherwise stated. All animal experiments were approved by Kindai University.

### 2.9 Colony forming assay

Cells were plated in MethoCult GF M3534 (STEMCELL Technologies, Inc.). Colony numbers were counted and cells were collected, pooled, and replated at the indicated cell numbers per 35‐mm plate. For AE and AE9a colony replating assay, c‐Kit^+^ bone marrow (BM) cells were isolated by magnetic‐activated cell sorting (MACS) (Miltenyi Biotec) and then cultured in medium containing 100 ng/ml murine stem cell factor (SCF), 100 ng/ml murine thrombopoietin (TPO) and 20 ng/ml murine interleukin‐6 (IL‐6) (Peprotech). The cells were subsequently infected with a retrovirus vector (MigR1‐AE, Addgene plasmid #12431; MigR1‐AE9a, Addgene plasmid #12433, respectively) twice, with a 24‐h interval. Flow cytometry‐sorted EGFP^+^ cells were used for further experiments. For RNA‐sequencing (RNA‐seq), RNAs were extracted from colony forming cells of the first plating round.

### 2.10 Mouse leukemia model

For primary transplantation experiments, 1 × 10^6^ AE9a‐transduced c‐Kit^+^ cells were administered intravenously by tail‐vein injection into 6‐week‐old female C57BL/6 mice irradiated with a dose of 10 Gy. For secondary transplantation experiments, sub‐lethally irradiated (8 Gy) female C57BL/6 recipient mice were injected with 4 × 10^5^ cells from leukemic spleen of the first recipients per mouse. All recipients received antibiotics‐water (fluconazole, enrofloxacin, neomycin) from 7 days before until 2 weeks after BM transplantation.

### 2.11 Statistics

Survival curves were constructed using Kaplan–Meier methodology and compared by the log‐rank test. Other statistical analyses were performed using the Student’s *t*‐test unless otherwise indicated.

## 3 RESULTS

### 3.1 *KDM4B* is elevated in patients with AML associated with t(8;21)

**FIGURE 1:**
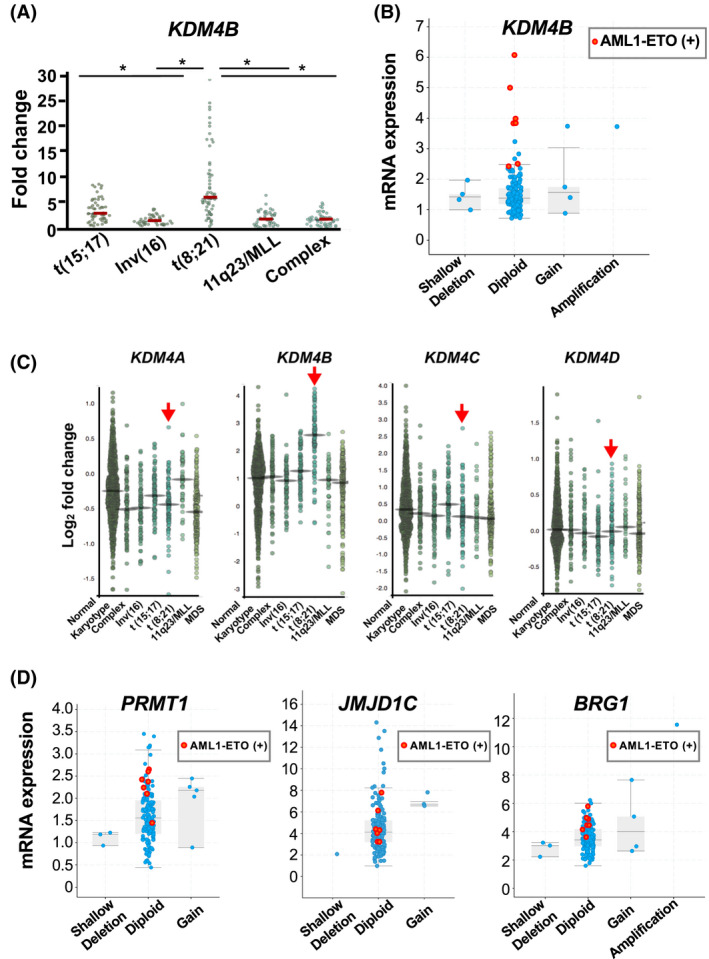
KDM4B is elevated specifically in AML associated with t(8;21). (A, B) Elevated *KDM4B* expression in human t(8;21) AML. Gene expression data from GSE6891 (A) and TCGA‐AML (B) were analyzed using BloodSpot (A) and cBioPortal (B), respectively. The red horizontal bars in (A) indicate the median values (*p < 0.001). The cases with *AML1*‐*ETO* fusion gene are indicated as orange dots in (B). The horizontal axis in (B) indicates putative copy‐number alterations from GISTIC. (C) *KDM4A–KDM4D* expression in different cytogenetic groups of human AML and MDS. Red arrows indicate t(8;21) AML. The data from GSE13159, GSE15434, GSE61804, GSE14468, and TCGA‐AML were analyzed using BloodSpot. MDS, myelodysplastic syndromes. (D) *PRMT1*, *JMJD1C*, and *BRG1 (SMARCA4)* expression in human AML. The data were analyzed using cBioPortal. The *AML1*‐*ETO*‐positive cases were indicated as orange dots. Neither significant co‐occurrence nor mutual exclusivity was detected (Fisher's exact test with *p* > 0.05) [Colour figure can be viewed at wileyonlinelibrary.com]

To investigate alterations in the expression of epigenetic molecules in leukemia, we initially searched gene expression profiles established from clinical samples, using an existing data portal for hematologic malignancies.^25^ As a result, *KDM4B* was identified as a gene that is variably but significantly highly expressed in AML cases associated with t(8;21) [hereafter referred to as t(8;21) AML]^26^ (Figure 1A). This result was confirmed by an analysis of The Cancer Genome Atlas AML (TCGA‐AML) cohort (Log_2_ odds ratio 4.842 and Fisher’s exact test with *p* < 0.0001)^27^, ^28^ (Figure 1B) as well as combined analyses of gene expression data from multiple studies^25^ (Figure 1C). Other KDM4 family members (i.e., *KDM4A*, *KDM4C*, and *KDM4D*) were found to be expressed at lower and similar levels across the cytogenetic subgroups of AML including the mixed lineage leukemia gene‐associated leukemia (11q23/MLL) etc. (Figure 1C). To date, several studies have indicated that histone modifiers and chromatin remodelers, such as protein arginine methyltransferase 1 (*PRMT1*), jumonji domain containing 1C (*JMJD1C)*, and SWI/SNF related, matrix associated, actin‐dependent regulator of chromatin, subfamily a, member 4 (*SMARCA4* also known as *BRG1*) facilitate the development of AML, including AE‐mediated leukemia.^29^, ^30^, ^31^ *KDM4B*, unlike these members (Figure 1D), is specifically elevated in t(8;21) AML, which prompted us in examining the role of KDM4B in this cytogenetic abnormality (Figure 1A–C).

### 3.2 KDM4B positively regulates cell proliferation in t(8;21) AML cell line models

**FIGURE 2:**
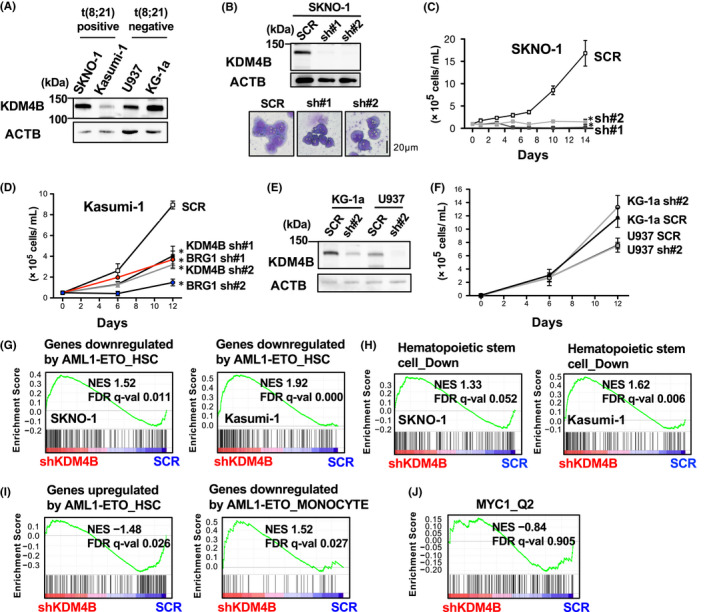
KDM4B silencing reduces cell proliferation in t(8;21)‐positive AML cell lines, coupled with impaired AML‐ETO‐mediated gene regulation and HSC gene signature. (A) Immunoblot showing KDM4B protein expression in AML cell lines using an anti‐KDM4B antibody. ACTB (β‐actin) was used as a loading control. (B) Immunoblot showing decreased KDM4B protein levels in SKNO‐1 cells stably infected with either of the two different shKDM4B (sh#1, sh#2) lentiviruses (top) and Wright–Giemsa‐stained cytospins of SKNO‐1 (bottom). (C) Anti‐proliferative effect of KDM4B silencing in t(8;21)‐positive and KDM4B‐high SKNO‐1 cells. Control shRNA (SCR), KDM4B sh#1‐, or sh#2‐transduced cells were seeded, and the cell number was counted on the indicated days. Error bars indicate SD. *p < 0.001. (D) Growth curves of t(8;21)‐positive and KDM4B‐low Kasumi‐1 cell line upon KDM4B and BRG1 silencing, respectively. Error bars indicate SD. *p < 0.005. (E) Immunoblot showing KDM4B protein expression in KG‐1a and U937 cells. ACTB (β‐actin) was used as a loading control. (F) Growth curves showing no significant growth suppressive effect of KDM4B silencing on t(8;21)‐negative KG‐1a and U937 cells. (G) GSEA plot showing a significantly increased expression of AML1‐ETO‐downregulated genes of human HSC (Genes downregulated by AML1‐ETO_HSC) in KDM4B‐silenced SKNO‐1 (left) and Kasumi‐1 (right). FDR, false discovery rate; NES, normalized enrichment score; SCR, control shRNA; shKDM4B, KDM4B sh#1 and sh#2. (H) GSEA plot showing a significant upregulation of human HSC under‐expressed genes (Hematopoietic stem cell_Down) in KDM4B‐silenced SKNO‐1 (left) and Kasumi‐1 (right). (I) GSEA plot for SKNO‐1 showing a decreased expression of AE‐upregulated genes of human HSCs (Genes upregulated by AML1‐ETO_HSC) (left) and an increased expression of AE‐downregulated genes of human monocytes (Genes downregulated by AML1‐ETO_MONOCYTE) (right) by KDM4B silencing. (J) GSEA plot showing comparable expression of MYC‐targets (MYC1_Q2 from MSigDB) in KDM4B‐silenced SKNO‐1 and SCR‐transduced control cells. GSEA, gene set enrichment analysis; HSC, hematopoietic stem cell; SD, standard deviation [Colour figure can be viewed at wileyonlinelibrary.com]

We then examined KDM4B protein expression levels in human AML cell lines, including two available t(8;21) AML‐derived SKNO‐1 and Kasumi‐1. The results indicated that SKNO‐1 has exhibited a higher level of the KDM4B protein compared with Kasumi‐1 (Figure 2A). In addition, among t(8;21)‐negative AML cell lines, we found that KDM4B protein was considerably expressed in the two U937 and KG‐1a cell lines (Figure 2A), which also may be useful to analyze the function of this molecule in AML. To examine the effect of KDM4B inhibition on t(8;21) AML, we first silenced the *KDM4B* gene in SKNO‐1 using a short hairpin RNA lentiviral system.^4^ Immunoblot analyses have confirmed the successful knockdown of KDM4B protein in the transduced cells (Figure 2B). KDM4B silencing did not significantly affect the total amount of di‐ and tri‐methylated H3K9 and tri‐methylated H3K36 levels (Figure S1A), which is consistent with the previous results from other studies performing KDM4B gene silencing in normally cultured cells^32^, ^33^ and may also be supported by the lower demethylase activity of KDM4B than the other KDM4 proteins.^3^ Notably, KDM4B silencing in SKNO‐1 induced a shrunken cell morphology and greatly reduced cell proliferation in a long‐term culture compared with the control (Figure 2B,C). This was coupled with increased cell death as determined by the AnnexinV^+^PI^−^ apoptotic fraction and trypan blue staining (not shown). KDM4B silencing exhibited a modest, but significantly growth‐suppressive effect on KDM4B‐lower expressed Kasumi‐1, which was comparable with the BRG1 silencing that was previously reported to attenuate Kasumi‐1 cell growth^31^ (Figure 2D). In contrast, KDM4B silencing conferred no significant growth suppression on KDM4B‐expressed U937 and KG‐1a cell lines until the cell density reached confluence (Figure 2E,F).

### 3.3 KDM4B retains AML1‐ETO‐target gene expression and undifferentiated gene expression patterns

To clarify the molecular mechanism of the anti‐proliferative effect of KDM4B silencing on t(8;21)‐positive cells, RNA‐seq was performed for the KDM4B‐silenced and control SKNO‐1 and Kasumi‐1, followed by GSEA applied to Hallmark gene sets, Kyoto Encyclopedia of Genes and Genomes pathways and gene ontology biological processes, but none of these gene sets were commonly changed in these two cell lines by KDM4B silencing at a significant level (FDR *q*‐value < 0.25). Previous studies have indicated that cell growth and survival of human t(8;21)‐positive AML cell lines are dependent on the *AE* fusion gene.^7^, ^34^ Therefore, we next determined the relevance of our gene expression data to AE‐inducible genes that are differentially expressed by the introduction of AE into primary human hematopoietic stem cells (HSCs).^15^ As a result, KDM4B silencing significantly increased the expression of AE‐downregulated genes (‘Genes downregulated by AML1‐ETO_HSC’) in both t(8;21)‐positive cell lines (Figure 2G), suggesting that the regulation of AE‐inducible gene expression is impaired by KDM4B inhibition. In addition, a significant upregulation of HSC under‐expressed genes (“Hematopoietic stem cell_Down”) was observed in both KDM4B‐silenced cell lines (Figure 2H), which may be associated with reduced undifferentiated properties.^17^ We then extracted subsets of genes that significantly contributed to the gene enrichment (called “core enrichment genes”, defined by GSEA) for the above two gene sets (Figure S1B). Indeed, 27 and 16 core enrichment genes were commonly upregulated in SKNO‐1 and Kasumi‐1 for the two gene sets, respectively, which included *OGG1* that is involved in base excision repair in AML1‐ETO‐mediated mutagenesis,^35^ tumor suppressors *RASSF2* ^36^ and *TGFBI*,^37^ and also *LAT2* that blocks differentiation in t(8;21)‐positive cells^38^ as repressive targets of AML1‐ETO. We further found that KDM4B silencing in SKNO‐1 but not Kasumi‐1 had inhibitory effects on the expression of a broader range of AE‐inducible genes (Figure 2I), which was compatible with an intensive growth‐suppressive effect following the gene silencing (Figure 2C). On the other hand, we saw no apparent compensatory expression of the other KDM4 family members, *KDM4A*, *KDM4C*, and *KDM4D* (not shown) or a significant global activation of MYC‐target genes which was previously reported to be positively regulated by KDM4B in neuroblastoma^39^ (Figure 2J). Collectively, KDM4B silencing in t(8;21)‐positive AML cell lines impairs the expression of AE‐inducible genes and genes associated with undifferentiated properties, thereby potentially contributing to the observed anti‐proliferative effect.

### 3.4 KDM4B‐dependent chromatin accessibility associates with AML1‐ETO target sites

**FIGURE 3:**
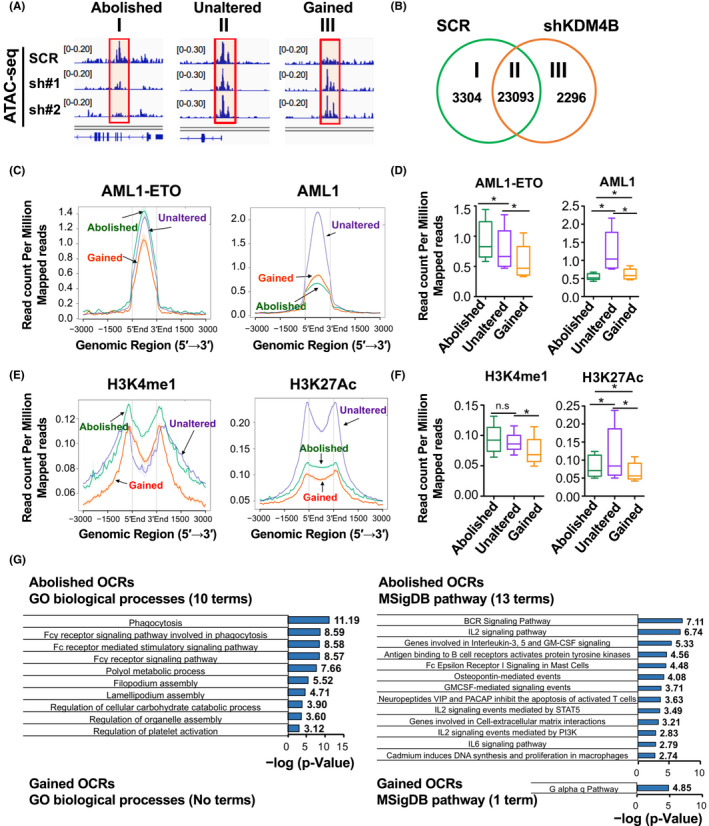
KDM4B preferentially regulates the chromatin accessibility of AML1‐ETO‐binding sites including enhancer histone mark deposition and functional gene regulatory regions. (A) Representative accessible chromatin sites identified using MACS2 peak caller following ATAC‐seq (I, II, and III, indicated by rectangles). I, SCR‐specific accessible chromatin sites (named abolished OCRs); II, accessible chromatin sites shared by SCR and KDM4B silencing (sh#1 and sh#2) (named unaltered OCRs); III, KDM4B silencing (sh#1 and sh#2)‐specific accessible chromatin sites (named gained OCRs). (B) Venn diagram showing the distribution of accessible chromatin sites of the three groups as indicated in (A). (C) ChIP signal distribution for AML1‐ETO (left) and AML1 (right) on the indicated accessible chromatin sites in SKNO‐1. (D) Box plot showing the “averaged read count per million mapped reads” values of the ChIP signal across the 5′ to 3′ ends of the accessible chromatin sites in (C), calculated using ngs.plot (line at median, Wilcoxon matched‐pairs signed‐rank test). *p < 0.0001. (E) ChIP signal distribution for enhancer histone marks H3K4me1 (left) and H3K27Ac (right) on the indicated accessible chromatin sites in SKNO‐1. (F) Box plot showing the “averaged read count per million mapped reads” values of the ChIP signal from −3000 to +3000 bp of the accessible chromatin sites in (E), calculated using ngs.plot (line at median, Wilcoxon matched‐pairs signed‐rank test). *p < 0.0001. (G) Bar graph showing the enrichment score (−log10[*p* value]) of significantly enriched annotations of GO biological processes (left) and MSigDB pathway (right) in accessible chromatin sites that were abolished and gained by KDM4B silencing. ChIP, chromatin immunoprecipitation; GO, gene ontology; OCR, open chromatin region [Colour figure can be viewed at wileyonlinelibrary.com]

Next, to examine the chromatin structure altered by KDM4B silencing, we analyzed KDM4B‐dependent chromatin accessibility using KDM4B‐high SKNO‐1, by assay for transposase‐accessible chromatin with sequencing (ATAC‐seq). KDM4B silencing redistributed accessible chromatin sites as expected, and we divided the identified open chromatin regions (OCRs) into three groups: (i) OCRs abolished by KDM4B silencing (control shRNA‐specific OCRs), (ii) unaltered OCRs, and (iii) OCRs gained by KDM4B silencing (KDM4B sh#1‐ and sh#2‐specific OCRs) (Figure 3A,B). By aligning the published chromatin immunoprecipitation sequencing (ChIP‐seq) signals for SKNO‐1^20^, ^21^ onto the three chromatin regions described above, we were able to determine that KDM4B silencing contributed to closing the chromatin regions where the AE protein significantly highly interacts (abolished, in Figure 3C, left, and Figure 3D, left). This also rendered a subset of AE‐binding regions aberrantly accessible (gained, in Figure 3C, left), accompanied by the reduction of AE protein entry (Figure 3D, left). Conversely, the binding of AML1 was primarily observed on the unaltered accessible regions (Figure 3C, right) and slightly rather higher on the gained OCRs compared with that on the abolished OCRs (Figure 3D, right). Thus, KDM4B silencing more preferentially affects chromatin accessibility of the AE‐target sites compared to that of the native AML1‐binding regions. Concurrently, the active enhancer hallmarks, mono‐methylated H3K4 (H3K4me1) and acetylated H3K27 (H3K27Ac), were also observed to be redistributed and deposited less on the accessible sites by KDM4B silencing (Figure 3E,F). These changes were accompanied by significantly decreased chromatin accessibility of cis‐acting DNA elements associated with multiple pathways, including myeloid signaling (Figure 3G), as analyzed by the Genomic Regions Enrichment of Annotations Tool.^23^ Thus, the chromatin accessibility of AE‐target sites is preferentially regulated by KDM4B, including enhancer histone mark deposition and functional gene regulatory regions, in the high KDM4B condition with endogenous AE.

### 3.5 Histone‐binding module of KDM4B is required for a proliferative surge in SKNO‐1 cells

**FIGURE 4:**
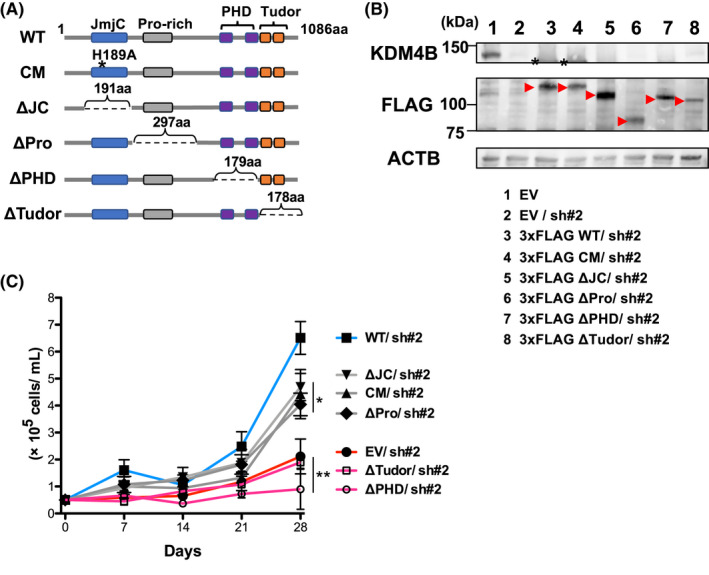
Murine KDM4B mutants lacking double PHD or Tudor domain do not confer a proliferative surge on the human KDM4B silencing in SKNO‐1 cells. (A) Domain structure of murine KDM4B proteins encoded by the wild‐type (WT) and mutant *Kdm4b* cDNA used in this study. The WT murine KDM4B protein (NCBI RefSeq: NP_742144) consists of 1086 amino acids. A triple FLAG tag sequence (3xFLAG) was introduced after the ATG starting codon. The regions indicated by dotted lines were deleted. The position of His189Ala is indicated by an asterisk. aa, amino acids; CM, catalytic mutant; Pro‐rich, proline‐rich region; PHD, plant homeodomain. (B) Immunoblot showing KDM4B silencing (sh#2) upon the expression of the triple FLAG (3xFLAG)‐tagged murine wild‐type or a series of mutated KDM4B proteins in SKNO‐1 cells. Asterisks indicate bands derived from the transduced murine proteins. Red arrowheads indicate positions of the transduced 3xFLAG‐tagged proteins as shown in (A). EV, empty vector. (C) Growth curves of the indicated transduced SKNO‐1 cells. SKNO‐1 cells were transduced with empty vector or a series of mutated *Kdm4b* cDNA in (A), followed by KDM4B knockdown using shRNA #2 against human *KDM4B*. Error bars indicate SD. *p < 0.05 (vs. EV/ sh#2, ΔTudor/sh#2, ΔPHD/sh#2 and WT/sh#2, respectively); **p < 0.005 (vs. WT/ sh#2). SD, standard deviation [Colour figure can be viewed at wileyonlinelibrary.com]

Recent studies have revealed non‐catalytic and catalytic function of histone methyltransferases and demethylases.^40^, ^41^ We then sought to elucidate the functional domains of KDM4B required for cell proliferation. Since KDM4B silencing transiently activated cell death, we therefore transduced SKNO‐1 cells with the empty vector, murine WT *Kdm4b* cDNA (78.7% identity and 84.9% similarity of amino acids to the human orthologue), a catalytic inactive mutant (H189A) and a *Kdm4b* mutant cDNA lacking JmjC domain, a proline rich‐domain covering region (Pro‐rich region), double PHD domain or double Tudor domain, respectively (Figure 4A), which was followed by KDM4B knockdown using shRNA #2 against human *KDM4B* (Figure S1C). This approach successfully achieved endogenous KDM4B silencing under the expression of the exogenous murine WT or mutated protein series (Figure 4B). Although this experimental system has induced a proliferative surge at a later time point compared to parental SKNO‐1 cells, we found that the transduction of murine WT, H189A and the JmjC‐deficient mutant conferred growth advantage on KDM4B‐silenced vector controls (Figure 4C). The exogenous expression of either PHD domain‐deficient or Tudor domain‐deficient mutant had a similar effect to that of the empty vector in KDM4B silencing (Figure 4C), suggesting that the histone‐binding module rather than the histone demethylase activity may contribute to the cell proliferation of SKNO‐1.

### 3.6 KDM4B promotes the development of AML1‐ETO‐induced leukemia in a mouse model

**FIGURE 5:**
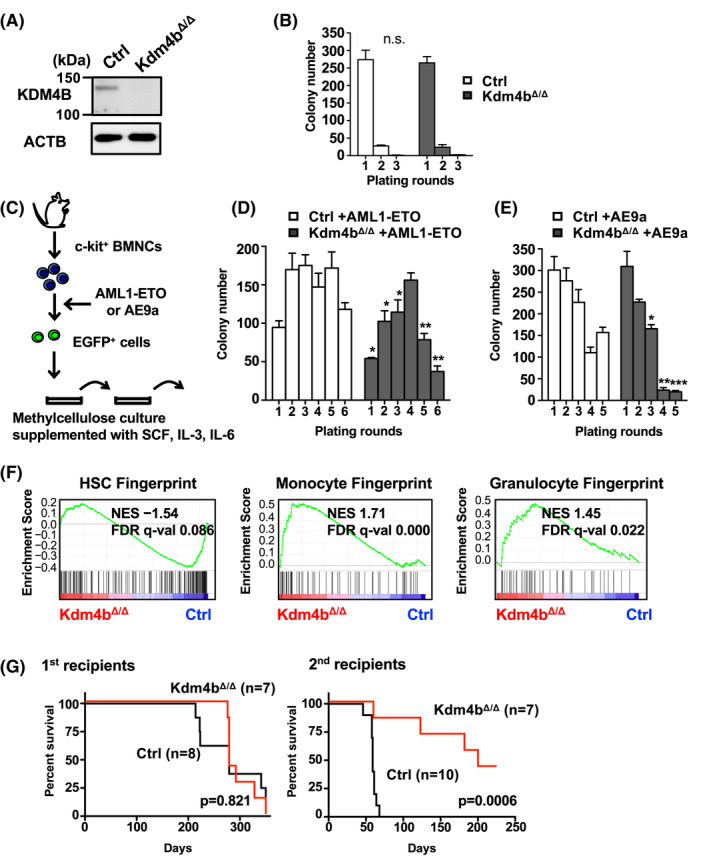
*Kdm4b* deficiency in mice reduces colony‐replating capacity and delays leukemia development associated with t(8;21) fusion genes. (A) Immunoblot showing decreased amounts of KDM4B protein in bone marrow mononucleated cells in *Kdm4b* *^Δ/Δ^* mice. (B) Comparable colony‐forming ability of c‐Kit^+^ HSC/progenitor cells from *Ctrl* or *Kdm4b^Δ^* ^/^ *^Δ^* mice in methylcellulose culture (MethoCult GF M3534). c‐Kit^+^ cells were plated initially at 1 × 10^4^ cells per plate. Colonies were counted 7 days post‐plating and then replated at 1 × 10^4^ cells every 7 days. Error bars indicate SD. n.s., not significant. (C) Schematic illustration of the colony‐replating assay procedure for (D, E). (D, E) Reduced colony‐replating capacity of *Kdm4b^Δ^* ^/^ *^Δ^* c‐Kit^+^ HSC/progenitor cells transduced with AML1‐ETO (D) and AE9a (E). The cells were initially plated at 1 × 10^4^. Colonies were then counted 7 days post‐plating and then replated at 2 × 10^4^ cells every 7 days. Error bars indicate SD. *p < 0.05; **p < 0.005; ***p < 0.0005. n.s., not significant. (F) GSEA plot showing significant downregulation of HSC‐fingerprint genes and upregulation of differentiated genes in AML1‐ETO‐transduced *Kdm4b^Δ^* ^/^ *^Δ^* colony‐forming cells. The plots for hematopoietic fingerprint genes of mouse HSC (HSC Fingerprint) (left), monocyte (Monocyte Fingerprint) (middle), and granulocyte (Granulocyte Fingerprint) (right) populations are shown. NES, normalized enrichment score; FDR, false discovery rate. (G) Impaired leukemia progression in the *Kdm4b* deficiency model. Kaplan–Meier survival curves of mice transplanted with AE9a‐transduced *Ctrl* or *Kdm4b^Δ^* ^/^ *^Δ^* cells in primary (left) [median survival: Ctrl = 279 days (*n* = 8), *Kdm4b^Δ^* ^/^ *^Δ^* = 279 days (*n* = 7); *p* = 0.821 (log‐rank test)] and secondary (right) [median survival: Ctrl = 59 days (*n* = 10), *Kdm4b^Δ^* ^/^ *^Δ^* = 200 days (*n* = 7); *p* = 0.0006 (log‐rank test)] transplantation assays. HSC, hematopoietic stem cell; SD, standard deviation [Colour figure can be viewed at wileyonlinelibrary.com]

To further explore the role of KDM4B in the development of leukemia, we established a polyinosinic‐polycytidylic acid (pIpC)‐inducible *Kdm4b*‐conditional knockout mouse model.^4^, ^24^ The *Mx1*‐*Cre*; *Kdm4b^flox^* ^/^ *^flox^* mice and littermate controls (*Kdm4b^flox^* ^/^ *^flox^*) were then injected with pIpC to induce Cre expression (hereafter referred to as *Kdm4b^Δ^* ^/^ *^Δ^* and *Ctrl* mice, respectively), and the resulting *Kdm4b^Δ^* ^/^ *^Δ^* mice exhibited a substantially decreased amount of KDM4B protein in BM nucleated cells (BMNCs) (Figure 5A). The *Kdm4b^Δ^* ^/^ *^Δ^* and *Ctrl* mice showed similar absolute cell numbers in subpopulations of hematopoietic stem/progenitor cells (HSPCs) in the BM (Figure S2A,B). In addition, c‐Kit^+^ BMNCs containing HSPCs from these mice exhibited comparable hematopoietic colony‐forming capacity in semi‐solid media (Figure 5B). The *Kdm4b^Δ^* ^/^ *^Δ^* and *Ctrl* mice have also displayed comparable cell populations in their peripheral blood at younger and older ages (3 weeks and 12 months post‐pIpC injection, respectively) (Figure S2C–E), suggesting that the conditional *Kdm4b* deletion does not affect hematopoiesis under normal conditions. Next, the c‐Kit^+^ BMNCs isolated from *Kdm4b^Δ^* ^/^ *^Δ^* and *Ctrl* mice were also retrovirally transduced with AE and AML1‐ETO9a (AE9a), a leukemia‐potent, C‐terminal‐deleted, truncated, splicing isoform of *AE*. The overexpression of either fusion gene into *Ctrl* cells enabled hematopoietic colonies to be serially replated in methylcellulose culture with cytokines (Figure 5C–E) as demonstrated previously.^42^, ^43^ In contrast, *Kdm4b* deficiency significantly reduced colony‐forming activity in successive rounds of plating (Figure 5D,E; Figure S3A), indicating a cell‐intrinsic requirement of *Kdm4b* for robust clonogenic potential coupled with t(8;21). Supporting the compromised colony‐forming properties in *Kdm4b* deficiency, AE‐transduced *Kdm4b^Δ^* ^/^ *^Δ^* colony‐forming cells displayed significantly decreased expression of HSC‐fingerprint genes, concurrently with increased expression of genes associated with differentiated states (monocyte and granulocyte fingerprints)^16^ (Figure 5F). Although AE is the gene primarily responsible for t(8;21) AML, transduction of AE into hematopoietic progenitors with BM transplantation has been reported not to induce leukemia in mice. Therefore, we instead injected AE9a‐transduced c‐Kit^+^ BMNCs from *Kdm4b^Δ^* ^/^ *^Δ^* or *Ctrl* mice into irradiated recipients for a serial BM transplant, which is often used for *in vivo* t(8;21)‐positive leukemia mouse models.^21^, ^44^, ^45^, ^46^ The recipient mice from both groups developed leukemia after 10 months (Figure 5G left), accompanied by the same type of splenic and peripheral blood leukemic cells (c‐Kit^−^Lin^−^ and/or c‐Kit^+^Lin^−^) as has been previously described.^47^ We however observed that AE9a‐transduced *Kdm4b^Δ^* ^/^ *^Δ^* cells exhibited a significant delay in leukemia development with a lower penetration and prolonged latency compared with AE9a‐transduced controls following transplant into secondary recipients, in which (AE9a‐IRES‐) EGFP levels in leukemic cells were comparably observed (Figure 5G right; Figure S3B,C). These results suggest that endogenous KDM4B enhances the development of AML *in vivo*.

## 4 DISCUSSION

Epigenetic alterations are among the most frequently observed aberrations in cancer.^48^ Concurrently, these alterations often alter the expression of a large number of genes, and accumulating evidence indicates that resulting net changes from epigenetic alterations are inevitably context‐ and cell type‐dependent.^48^ An example is that a polycomb group protein CBX7 is oncogenic in hematological malignancies but plays tumor‐suppressive roles in certain epithelial‐based tumor contexts.^49^ Also, a histone methyltransferase EZH2 was recently reported to show opposing (oncogenic and tumor‐suppressive) roles during the initiation and maintenance of the same AML.^50^ Thus, in what cellular context epigenetic mechanisms could drive the tumor development is obviously considered to be a key issue in designing therapeutic strategies.

In this study, we demonstrated that KDM4B has a tumor‐promoting role in AE‐induced leukemia, using human cell line models and mouse genetic ablation. Stable suppression of KDM4B resulted in a substantial decrease in cell proliferation in t(8;21)‐positive SKNO‐1 and Kasumi‐1 cell lines. Consistently, murine *Kdm4b* deficiency reduced clonogenic activity of hematopoietic progenitor cells transduced with AE and AE9a, and impaired leukemia development in a mouse BMT model. A previous study revealed that combined activity of *Kdm4a*, *Kdm4b*, and *Kdm4c* is required for AML with a rearrangement of the mixed‐lineage leukemia gene (MLL‐AF9‐translocated AML) *in vitro* and *in vivo*, using *Kdm4a*−*c* triple knockout mice.^51^ The authors identified *interleukin 3 receptor α (IL3ra)* as a critical target gene downstream of those KDM4 proteins in promoting AML.^51^ The present results of our experiments show, for the first time, that KDM4B alone plays a significant role in promoting leukemia, in the context of t(8;21)‐positive AML. Although we have already examined our RNA‐seq results of human cell lines and mouse hematopoietic cells obtained in this present study, *IL3RA*/*IL3ra* gene expression was not significantly altered by KDM4B single deficiency in the presence of AE. Alternatively, we discovered that KDM4B silencing impaired the expression of AE‐inducible genes and HSC signature in AE‐expressing cells. KDM4B silencing also did not significantly affect proliferation of t(8;21)‐negative U937 and KG‐1a cell lines, suggesting that the requirement of KDM4B may be linked to the context of AE‐induced leukemia. Supporting this idea, the chromatin accessibility of AE‐binding sites was revealed to be more preferentially regulated by KDM4B compared to that of native AML1‐binding sites, involving active enhancer marks and functional cis‐regulatory elements, which was observed using a t(8;21)‐derived AML cell model and demonstrated by open chromatin analysis. Since AE is a key driver for t(8;21)‐positive leukemia, the results from our study may partly explain the mechanism of anti‐leukemic activities of KDM4B inhibition. The transduction of murine WT or a series of mutated KDM4B proteins, followed by human KDM4B silencing, demonstrated that the double PHD or double Tudor domain rather than the catalytic function may contribute to the cell growth of SKNO‐1 cells. These methylated‐histone binding modules potentially act as scaffolds that recruit various components, including chromatin modifiers to genomic regions.^52^ Further studies will be required to elucidate how KDM4B silencing induces changes in the chromatin occupancy and enhancer modifications leading to anti‐leukemic activities.

The mechanism underlying *KDM4B* gene upregulation in t(8;21) AML remains to be clarified. The AE protein has been determined to bind to a promoter region, including the transcription start site (TSS) of the human *KDM4B* gene in SKNO‐1 and Kasumi‐1 cells, on the basis of an analysis on the published ChIP‐seq results^20^, ^53^ (Figure S1D). We further found AE (and AML1)‐binding consensus (Py‐G‐Py‐GGT‐Py) sequences in this region (Figure S1D), suggesting that *KDM4B* might be a direct transcriptional target of AE. To test this possibility, we examined the promoter activity of a *KDM4B* gene fragment covering the TSS (from −900 to +100 bp), using a luciferase assay by transducing AE into HEK293 cells in combination with or without AML1 and/or the CBFB cofactor. However, no clear promoter activation has been noted, at least in this experimental system. We thus speculate that *KDM4B* gene upregulation may require additional factors; otherwise, it may be a result of clonal selection with genetic interaction in which KDM4B provides an AE‐preferential chromatin state.

Studies have so far revealed that the inhibition of *PRMT1 and SMARCA4*, respectively, suppressed the leukemic activities in AE‐positive and other types of AML cells.^29^, ^31^, ^54^ In addition, a recent prior unbiased proteomic analysis of AE‐associated proteins identified a JmjC domain‐containing JMJD1C as a component that physically interacts with AE to facilitate AE‐induced leukemia.^30^ The authors showed that JMJD1C also interacts with several other leukemic transcription factors and that this molecule is required for the survival of a great variety of AML but not only t(8;21) AML. Although KDM4B was not included as a component of major AE‐containing protein complexes according to the study, our results nevertheless show a certain genetic interaction between KDM4B and AML1‐ETO in promoting AML, and the gene expression analysis and ATAC‐seq in this study also support the functional involvement of KDM4B in transcriptional regulation mediated by AML‐ETO, suggesting that KDM4B presumably indirectly interacts with AE. Delineating the connection between KDM4B and the aforementioned reported epigenetic regulators will promote a better understanding of epigenetic regulation in AE‐induced leukemia, and facilitate the development of novel therapies to improve the treatment outcome.

## CONFLICT OF INTERESTS

The authors declare no competing financial interests.

## AUTHOR CONTRIBUTIONS

Takeshi Ueda and Hitoshi Okada designed the research. Takeshi Ueda, Akinori Kanai, Akiyoshi Komuro, Hisayuki Amano, Kazushige Ota, Masahiko Honda, Masahito Kawazu, and Hitoshi Okada performed the research. Takeshi Ueda, Akinori Kanai, and Hitoshi Okada analyzed the data. Takeshi Ueda and Hitoshi Okada wrote the manuscript. and all authors read and approved the final manuscript.

## Supporting information

Supplementary Material

## Data Availability

RNA‐seq data and ATAC‐seq data obtained in this study have been deposited under the following accession numbers: Experiment DRX227431–DRX227443 in the DDBJ (DNA Data Bank of Japan).
