# Supplementary material for: KDM4B promotes acute myeloid leukemia associated with AML1‐ETO by regulating chromatin accessibility
Source: FASEB Bioadv. 2021 Sep 12;3(12):1020–33. doi: 10.1096/fba.2021-00030 (PMC8664044; doi:10.1096/fba.2021-00030)
Supplement: Supplementary file 1 — Supplementary Material [file FBA2-3-1020-s001.pdf]

Supporting Information

Supplemental Materials and Methods

Surface marker analysis by flow cytometry. Surface marker phenotypes to separate mouse stem and progenitor cell subsets were as follows: LT-HSC: $CD34^{-}$ $CD135^{-}$ $c\text{-}Kit^{+}$ $Sca\text{-}1^{+}$ Lineage $marker^{-}$; ST-HSC: $CD34^{+}$ $CD135^{-}$ $c\text{-}Kit^{+}$ $Sca\text{-}1^{+}$ Lineage $marker^{-}$; MPP: $CD34^{+}$ $CD135^{+}$ $c\text{-}Kit^{+}$ $Sca\text{-}1^{+}$ Lineage $marker^{-}$; CMP: $CD34^{+}$ $CD16/32^{low}$ $c\text{-}Kit^{+}$ $Sca\text{-}1^{-}$ Lineage $marker^{-}$; GMP: $CD34^{+}$ $CD16/32^{high}$ $c\text{-}Kit^{+}$ $Sca\text{-}1^{-}$ Lineage $marker^{-}$; MEP: $CD34^{-}$ $CD16/32^{-}$ $c\text{-}Kit^{+}$ $Sca\text{-}1^{-}$ Lineage $marker^{-}$. CD4, CD8, B220, Mac1, Gr1 and Ter119 were used to exclude lineage-committed cells. Cells were stained with fluorescence-conjugated antibodies against the following antigens: c-Kit (2B8), Sca-1(D7), CD34(RAM34), CD135(A2F10.1), CD16/CD32 (2.4G2), CD4 (GK1.5 or RM4-5), CD8(53-6.7), Gr-1(RB6-8C5), Mac1(M1/70), Thy1.2(30-H12) and B220 (RA3-6B2) and Ter119 (TER-119). All antibodies were from BD Biosciences, BioLegend or eBioscience. Data were acquired by either BD FACSAria, BD FACSCantoII or BD LSRFortessa flow cytometer and analyzed with the FlowJo analysis program.

Antibodies for immunoblotting

The following antibodies were used for immunoblotting: anti-KDM4B (JMJD2B) (A301-478A) (Bethyl Laboratories); anti-β-actin (sc-47778) (Santa Cruz Biotechnology) , anti-FLAG (anti-DDDDK tag) (PM020) (Medical & Biological Laboratories); anti-Histone H3 (#9715) and anti-tri-methyl-histone H3 (Lys36) (#4909) (Cell Signaling Technology); anti-histone H3 (tri methyl K9) (ab8898) (Abcam); anti-dimethyl-Histone H3 (Lys9) (07-441) (Upstate); and anti-rabbit IgG, HRP-linked whole antibody (NA934) and anti-mouse IgG, HRP-linked whole antibody (NA931) (Amersham).

Supplemental Figure S1

(A)

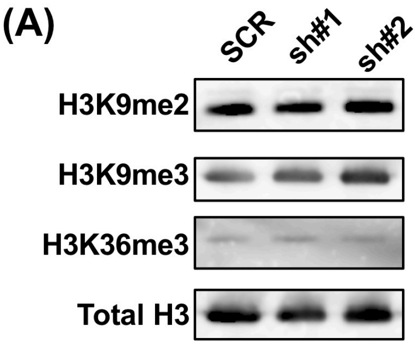

(B)

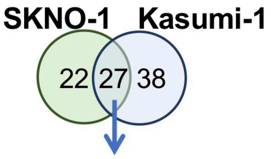

SLCO4C1
SLA
SELPLG
TGFBI
SLC48A1
LST1
OGG1
MICAL2
TPM1
BIN2
ITGA6
PLAC8
ZBTB16
SASH3
MYCN
CSTA
MS4A3
RPS6KA1
MBP
GSR
CKAP4
TMEM156
SLCO3A1
LAT2
TUBA4A
RASSF2
KBTBD11

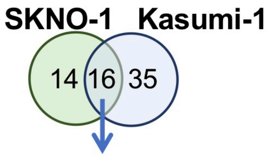

IGSF6
KLF2
TGFBI
LILRA5
CDK5R1
ABCC13
FGR
CSTA
F13A1
LYZ
NCF2
GABARAPL1
TUBB1
NPL
CTTN
HCK

(C)

Human *KDM4B* sh#1 target	G**G**AAGGACATGGTCAAGAT
Murine *Kdm4b* cDNA 929-947	G**A**AAGGACATGGTCAAGAT
Human *KDM4B* sh#2 target	G**G**TGGAA**G**CTGAAATGC**G**T
Murine *Kdm4b* cDNA 2504-2522	G**A**TGGAA**A**CTGAAATGC**A**T

(D)

Human chromosome 19

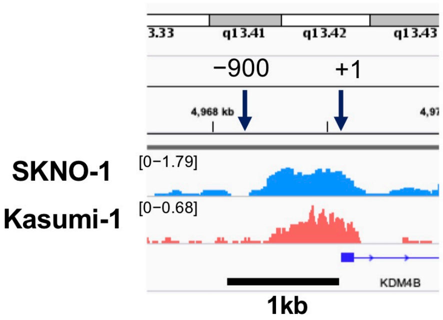

−900 actaccatcatatccattgcacagttggaaacagactcagcgaggt
tactttgctggaggtc**aaccaca**gtgaggcatggggcccggcttag
ctttgaatccaggtcagaaagcaccaaagctcattctctcccatac
attttagggaactcggggagagggaccctaactattccgaaggcct
tgccccttggcaaaggtttgttgattgtgatgggaatagacctacg
gtggcggggtggaggctcttgctgctttaccctatgttctcggttc
tcctcctttgcaaaacggatcgctatgtctccttgcttggcacttt
gcaaattggggaacgctaagcccacattccggccacgggtttcgaa
cctccagcctgcacgttcccggccggtgctgatgctgtgctgggtc
tcacgtactcagccgccgcctgataaccagggcggggcccggagct
tgcgggcagtgattggcacctgccccagcctgtccgcgcctcgggc
tggcccctcaagccaatactggctcctctctg**tgcggtc**gtcgggc
gggccctgaggctcccttgtcaatccaaggcccagctgccgtcggg
tt −301

Supplemental Figure S2

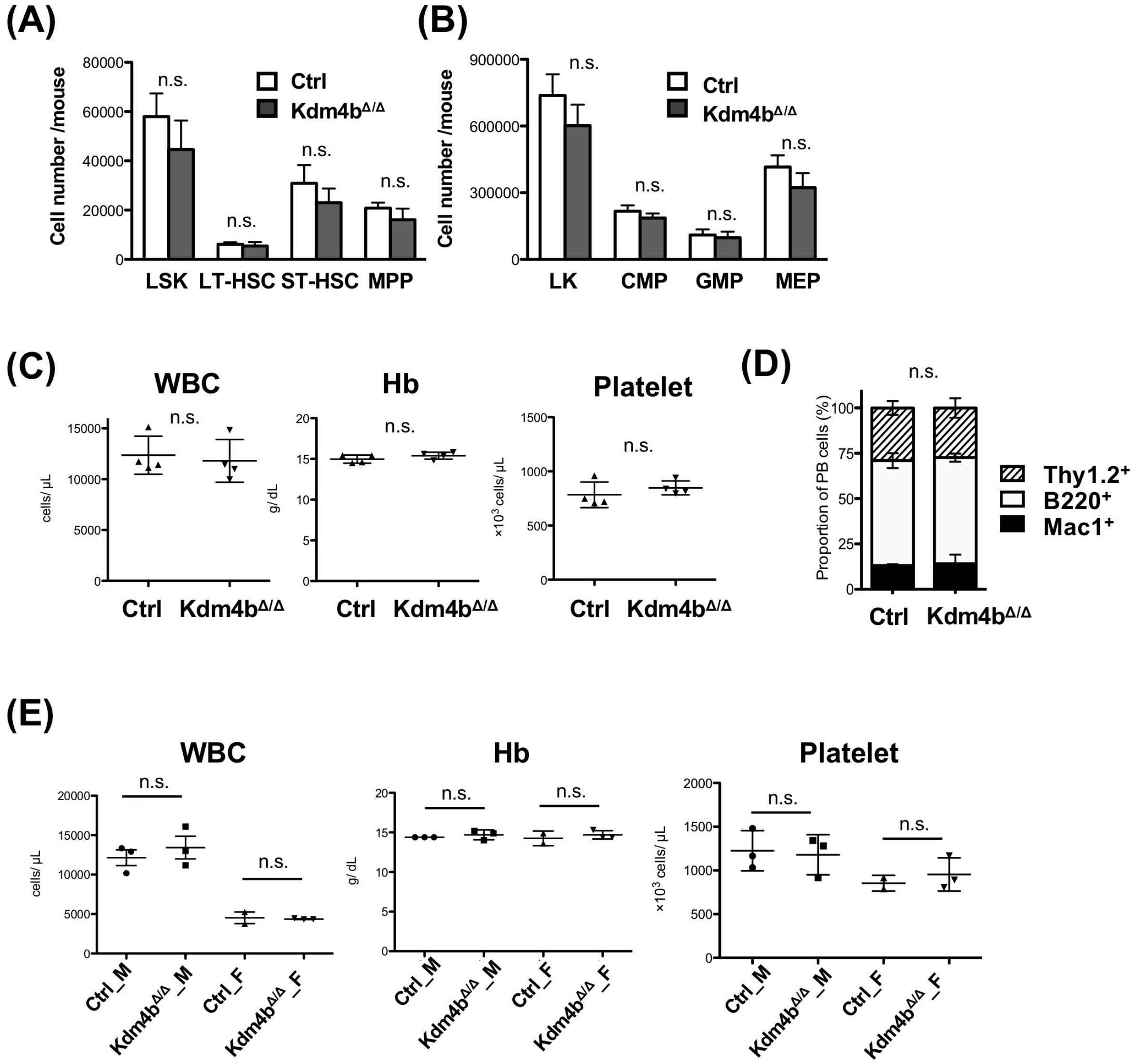

Supplemental Figure S3

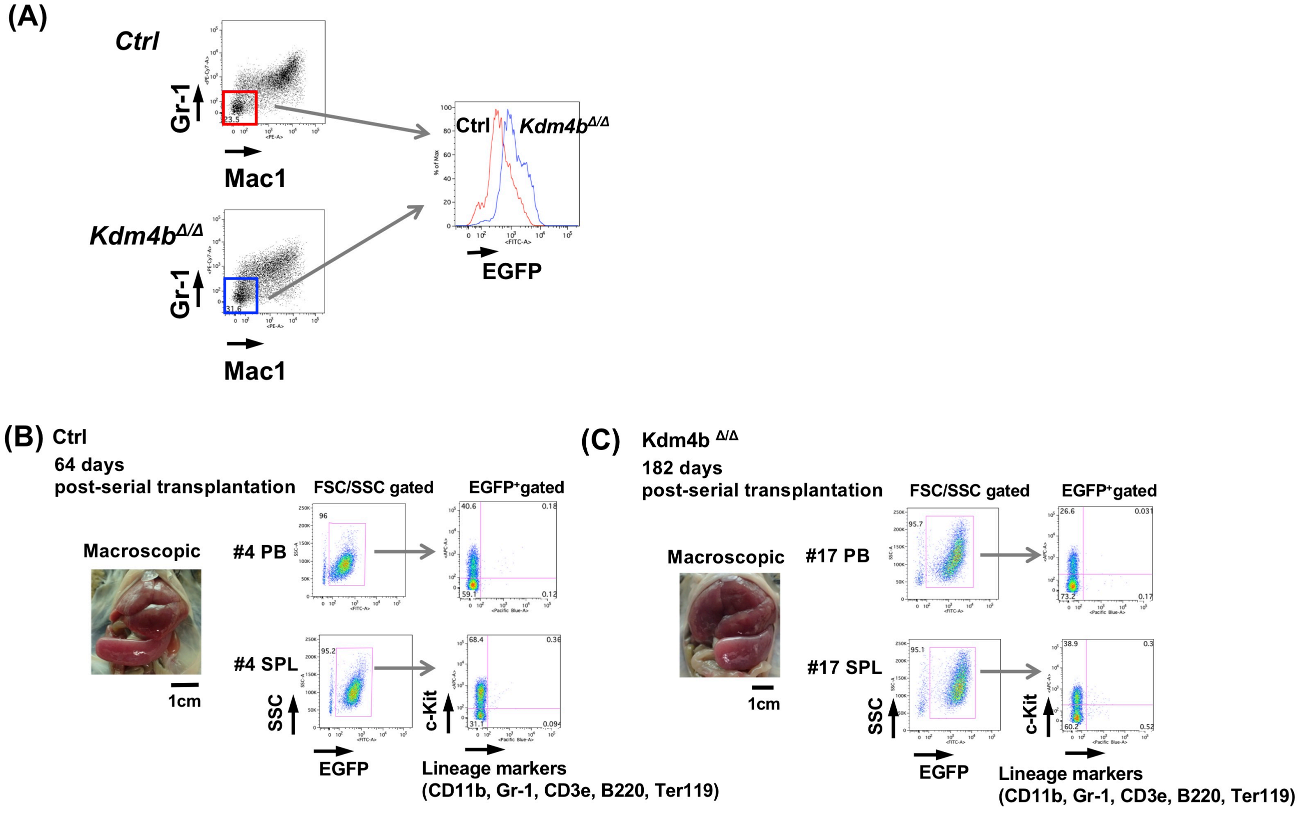

Supplemental Figure Legends

Supplemental Figure S1. Supplemental results from experiments using t(8;21) AML cell lines

(A) Immunoblot showing comparable di- and tri-methylated H3K9 (H3K9me2 and H3K9me3) and tri-methylated H3K36 (H3K36me3) levels in KDM4B-silenced SKNO-1 cells and the controls.

(B) Shared core enrichment genes in KDM4B-silenced SKNO-1 and Kasumi-1 cell lines for 'Genes downregulated by AML1-ETO_HSC' (left) and 'Hematopoietic stem cell_Down' (right), corresponding to Figure 2G and 2H.

(C) Comparison of human KDM4B sh#1 and sh#2 targets with partial sequences of murine *Kdm4b* cDNA. The alignment of human KDM4B sh#1 and sh#2 targets to murine *Kdm4b* cDNA nucleotide sequences with minimal mismatches is shown. One and three mismatched nucleotide sequences of human KDM4B sh#1 and sh#2 targets are found in the indicated positions of murine *Kdm4b* cDNA. Besides, six mismatched nucleotide sequences of KDM4B sh#1 and sh#2 targets are found in three and two positions of murine *Kdm4b* cDNA, respectively (not shown).

(D) AML1-ETO (AE) binds to a promoter region of the *KDM4B* gene in t(8;21)-positive AML cell lines. IGV snapshot showing the binding of AE to a promoter region of the *KDM4B* gene in t(8;21)-positive SKNO-1 and Kasumi-1 cell lines (data from SRX026638 and SRX236112) (left). An AE-binding consensus motif (Py-G-Py-GGT-Py) and its reverse-complementary sequences are found in the AE binding region (shown in bold with underline) (right).

Supplemental Figure S2. Supplemental results from experiments using *Kdm4b*$^{\Delta/\Delta}$ and *Ctrl* mice

(A) The numbers of bone marrow HSCs (LSK, LT-HSC, ST-HSC, and MPP) from *Kdm4b*$^{\Delta/\Delta}$ and *Ctrl* mice (n = 4 per group, males). LSK contains long-term HSC (LT-HSC), short-term HSC (ST-HSC) and multipotent progenitors (MPP). n.s., not significant.

(B) The numbers of bone marrow progenitor cells (LK, CMP, GMP and MEP) from *Kdm4b*$^{\Delta/\Delta}$ and *Ctrl* mice (n = 4 per group, males). LK population, c-Kit-positive hematopoietic progenitors negative for Sca-1 and lineage markers (c-Kit^{+}Sca-1^{-}Lin^{-}), contains common myeloid progenitor (CMP), granulocyte macrophage progenitor (GMP) and megakaryocyte/erythroid progenitor (MEP). n.s., not significant.

(C) The numbers of white blood cells (WBC) and platelets are shown, with the concentration of Hb in the peripheral blood from the same littermates (3 weeks post-pIpC treatment) (n = 4 per group, females). Horizontal bars indicate mean values. Error bars, SD; n.s., not significant.
(D) The ratio of myeloid (Mac1^{+}), B lymphoid (B220^{+}), and T lymphoid (Thy1.2^{+}) lineages to WBCs in (C) is shown. The mean ratios are presented. Error bars, SD; n.s., not significant.
(E) The numbers of white blood cells (WBC) and platelets are shown, with the concentration of Hb in the peripheral blood from the two littermates (12 months post-pIpC treatment). M, male; F, female. Horizontal bars indicate mean values. Error bars, SD; n.s., not significant.

Supplemental Figure S3. Flow cytometric detection of bicistronically-expressed EGFP fluorescence in combination with surface marker analysis for *Kdm4b*$^{\Delta/\Delta}$ and *Ctrl*-derived cells

(A) Flow cytometric plots for the detection of EGFP in AML1-ETO-transduced colony-forming cells. Colony-forming cells were harvested at the fifth plating round, and then the EGFP fluorescence levels were analyzed (right panel) in gated Gr-1/ Mac1 (differentiation marker)-negative fractions (left panels). *Kdm4b*-deficient colony-forming cells displayed rather higher levels of EGFP than do the controls at this time point.
(B and C) Representative macroscopic appearance (left of each figure) of leukemic recipient mice transplanted with AE9a-transduced *Ctrl* (B) and *Kdm4b*$^{\Delta/\Delta}$ (C) cells, respectively. Massive hepatosplenomegaly is shown. Flow cytometric analysis (right of each figure) of mice that developed AE9a-mediated leukemia. Note that EGFP^{+} cells are negative for lineage markers and partly positive for c-Kit, as consistent with the previously reported surface marker phenotype of the AE9a-mediated leukemia. PB, peripheral blood; SPL, spleen.
